# Supplementary material for: Nutritional Factors Modulating Alu Methylation in an Italian Sample from The Mark-Age Study Including Offspring of Healthy Nonagenarians
Source: Nutrients. 2019 Dec 6;11(12):2986. doi: 10.3390/nu11122986 (PMC6950565; doi:10.3390/nu11122986)
Supplement: Supplementary file 1 [file nutrients-11-02986-s001.pdf]

Article

Nutritional Factors Modulating Alu Methylation in an Italian Sample from The Mark-Age Study Including Offspring of Healthy Nonagenarians

Robertina Giacconi [1,†], Marco Malavolta [1,†], Alexander Bürkle [2], María Moreno-Villanueva [2,3], Claudio Franceschi [4], Miriam Capri [5], P. Eline Slagboom [6], Eugène H.J.M. Jansen [7], Martijn E. T. Dollé [7], Tilman Grune [8,9], Daniela Weber [8], Antti Hervonen [10], Wolfgang Stuetz [11], Nicolle Breusing [11], Fabio Ciccarone [12], Michele Zampieri [13,14], Valentina Aversano [13,14], Paola Caiafa [13,14], Laura Formentini [1], Francesco Piacenza [1], Elisa Pierpaoli [1], Andrea Basso [1], Mauro Provinciali [1,*] and Maurizio Cardelli [1]

[1] Advanced Technology Center for Aging Research, IRCCS INRCA, 60121 Ancona, Italy; r.giacconi@inrca.it (R.G.); m.malavolta@inrca.it (M.M.); lauraformentini26@gmail.com (L.F.); f.piacenza@inrca.it (F.P.); e.pierpaoli@inrca.it (E.P.); a.basso@inrca.it (A.B.); m.cardelli@inrca.it (M.C.)

[23] Molecular Toxicology Group, Department of Biology, Box 628, University of Konstanz, 78457 Konstanz, Germany; alexander.buerkle@uni-konstanz.de (A.B.); maria.moreno-villanueva@uni-konstanz.de (M.M.-V.)

[3] Human Performance Research Centre, Department of Sport Science, Box 30, University of Konstanz, 78457 Konstanz, Germany

[4]Lobachevsky State University of Nizhny Novgorod, 603950 city, Russia; claudio.franceschi@unibo.it

[5] DIMES-Department of Experimental, Diagnostic and Specialty Medicine, Via S. Giacomo, 12, ALMA MATER STUDIORUM, University of Bologna, 40126 Bologna, Italy; miriam.capri@unibo.it

[6] Section of Molecular Epidemiology, Leiden University Medical Centre, 2333 ZA Leiden, The Netherlands; p.slagboom@lumc.nl

[7] Centre for Health Protection, National Institute for Public Health and the Environment, PO Box 1, 3720 BA Bilthoven, The Netherlands; eugenejansen99@gmail.com (E.H.J.M.J.); martijn.dolle@rivm.nl (M.E.T.D.)

[8] Department of Molecular Toxicology, German Institute of Human Nutrition Potsdam-Rehbruecke (DIfE), 14558 Nuthetal, Germany; scientific.director@dife.de

[9] NutriAct-Competence Cluster Nutrition Research Berlin-Potsdam, Nuthetal 14458, Germany

[10] Medical School, University of Tampere, 33014 Tampere, Finland; antti.Hervonen@uta.fi

[11] Institute of Nutrition, University of Hohenheim, Stuttgart 70599, Germany; nicolle_breusing@yahoo.de

[12] 12 IRCCS San Raffaele Pisana, Department of Human Sciences and Promotion of the Quality of Life, San Raffaele Roma Open University, 00166 Rome, Italy; ciccarone@bio.uniroma2.it

[13] Faculty of Pharmacy and Medicine, Department of Experimental Medicine, Sapienza University of Rome, Rome 00161, Italy; michele.zampieri@uniroma1.it (M.Z.); aversano@bce.uniroma1.it (V.A.); caiafa@bce.uniroma1.it (P.C.)

[14] Pasteur Institute-Fondazione Cenci Bolognetti, Rome 00161, Italy

* Correspondence: m.provinciali@inrca.it; Tel.: +39-071-8004210; Fax: +39-071-206791

† These authors contributed equally to this work.

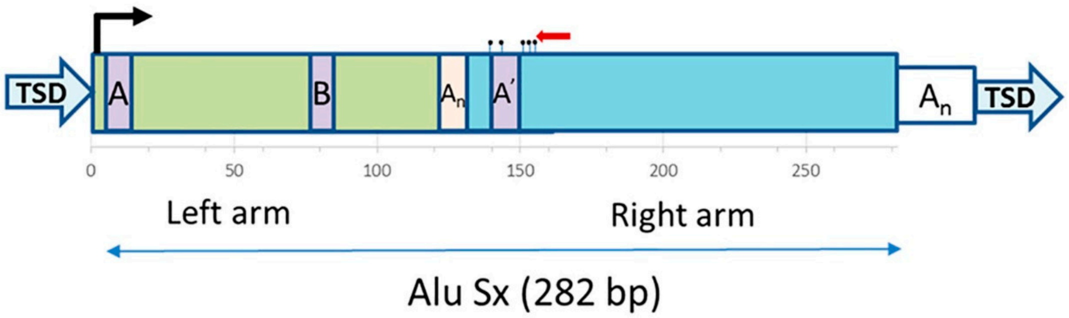

Supplementary Figure 1S. Schematic representation of an Alu element, composed by a left arm and a right arm, divided by an A-rich region (A_n in the center of the graph). The bended arrow on the left represents the transcription start site. A typical Alu element is followed by a poly-A tail (A_n on the right of the graph) and flanked two direct repeats (target site duplications, TSD in the graph). The other letters in the graph indicate the main transcription factor binding sites, known as A box, B box and A' box.

The five small "lollipops" above the central part of the graph indicate the position of the five CpG methylation sites which have been assayed by bisulfite pyrosequencing in this work. The small arrow on the right of the "lollipops" indicates the region corresponding to the pyrosequencing primer, and the sequencing direction. The scale, and the position of the sequence features in the scheme, are calculated based on a consensus sequence of Alu Sx subfamily, accessed through the Repbase Update database (Jurka et. al., 2005) on the Genetic Information Research Institute web site (www.girinst.org). The exact position of the internal features in the Alu Sx sequence is based on previous publications (Cui et al., 2011; Luo et al., 2014).

Supplementary Figure 2S. Position of the assayed CpGs in the Alu Sx consensus sequence (before and after bisulfite conversion)

```
1   GGCCGGGCGC GGTGGCTCAC GCCTGTAATC CCAGCACTTT GGGAGGCCGA GGCGGGCGGA TCACCTGAGG TCAGGAGTTC GAGACCAGCC TGGCCAACAT

101 GGTGAAACCC CGTCTCTACT AAAAATACAA AAATTAGCCG GGCGTGGTGG CGCGCGCCTG TAATCCCAGC TACTCGGGAG GCTGAGGCAG GAGAATCGCT

201 TGAACCCGGG AGGCGGAGGT TGCAGTGAGC CGAGATCGCG CCACTGCACT CCAGCCTGGG CGACAGAGCG AGACTCCGTC TCA
```

```
    TTTTTATTAA AAATATAAAA ATT
    |||||||||| |||||||||| |||
113 TTTTTATTAA AAATATAAAA ATTAGTYGGG YGTGGTGGYG YGYGTTTGTA ATTTTAGTTA TTYGGGAGGT TGAGGTAGGA GAATYGTTTG
                                                  ||||||| |||||||||| ||
                                                  CAAACAT TAAAATCAAT AA


203 AATTYGGGAG GYGGAGGTTG TAGTGAGTYG AGATYGYGTT ATTGTATTTT AGTTTGGG 261
                                             || |||||||||| ||||||||
                                             AA TAACATAAAA TCAAACCC
```

Upper box: the consensus sequence of Alu Sx subfamily, obtained through the Repbase Update database (Jurka et. al., 2005) available at the Genetic Information Research Institute web site (www.girinst.org). The region analysed in the bisulfite pyrosequencing assay is evidenced in blue. The letters in bold represent the 5 assayed CpGs. The sequencing direction is antisense with respect to the consensus, hence the CpG n.1 in the assay corresponds to the CpG located more near to the 3'-end in the above reported sequence. The underlined traits (starting from the 5' end) represent the following features of the Alu sequence: the A box; the B box; the A-rich region; the A' box (Cuo et al., 2011; Luo et al., 2014).

Lower box: the region of the bisulfite-converted Alu Sx consensus corresponding to the assay target is reported. Letters " Y" indicates CpG cytosines, which have been converted into thymines only when not methylated. The PCR primers and the sequencing primer are also shown. The box indicates the region assayed by pyrosequencing, the arrow represents the sequencing direction, and the letters evidenced in yellow represent the five CpG cytosines (partially converted in thymines) for which the percentage of methylation is measured.

Supplementary Table 1S

**Details of the pyrosequencing assay for Alu elements**
Sequence to analyze
RYRYRCCACYAYRCCYRACTAA
Dispensation order
CGACTGACTGACTACTCACTGACTAGACT
**Details of the pyroseqeuncing assay for PyroMark Control Oligo (Qiagen)**
TAYGGTTTGC
dispensation order
CTGACTGTG

Supplementary Figure 3S. Calibration curves obtained by pyrosequencing analysis of a control, bisulfite-converted genomic DNA sample. To generate each point of the curve, a sample with known methylation has been obtained by mixing different proportions of a totally methylated and a totally unmethylated converted genomic DNA sample (control samples obtained from Qiagen). *Note*: “True methylation”: known methylation of the control sample; “Estimated methylation”: methylation value obtained by the Alu pyrosequencing assay.

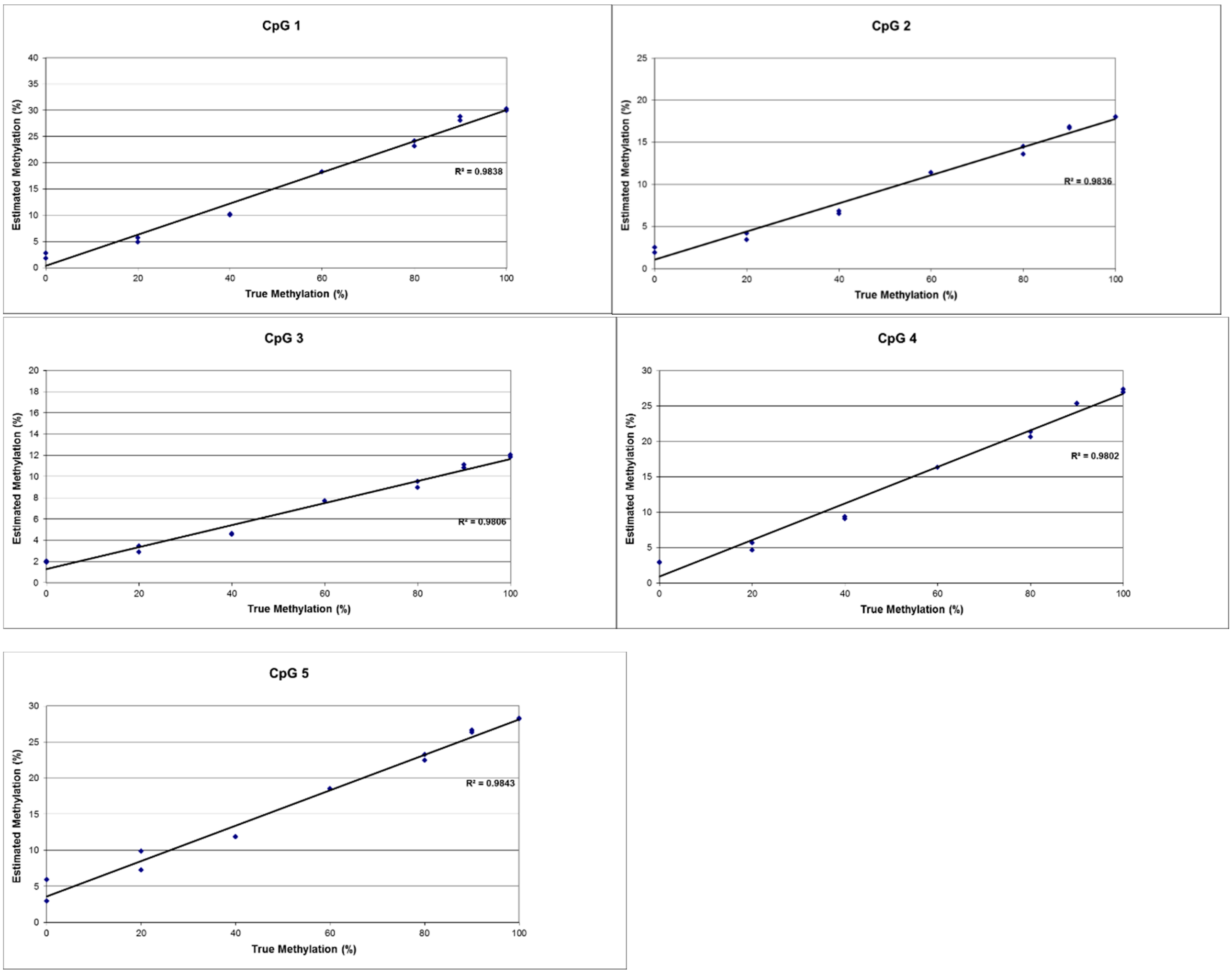

Supplementary Figure 4S. Alu bisulfite pyrosequencing: in silico determination of the assay targets

The complementarity of the primers used for the bisulfite pyrosequencing assay has been checked against the consensus sequences of all Alu subfamilies present in the RepBase Update database (www.girinst.org, data downloaded on 31-10-2017). Only part of the Alu S subfamilies have a complete complementarity (after bisulfite conversion) with all the primers (the two PCR primers and the pyrosequencing primer) used in the assay: Alu Sx, Alu Sg, Alu Sz, Alu Sz6, Alu Sg1, Alu Sg4, Alu Sq, Alu Sq4, Alu Sx1, Alu Sg7, Alu Sq2, Alu Sq10 and Alu Sp. As shown in the Supplemental figure below, only part of the Alu subfamilies targeted by the assay contain in their consensus sequences all the 5 CpG sites analysed.

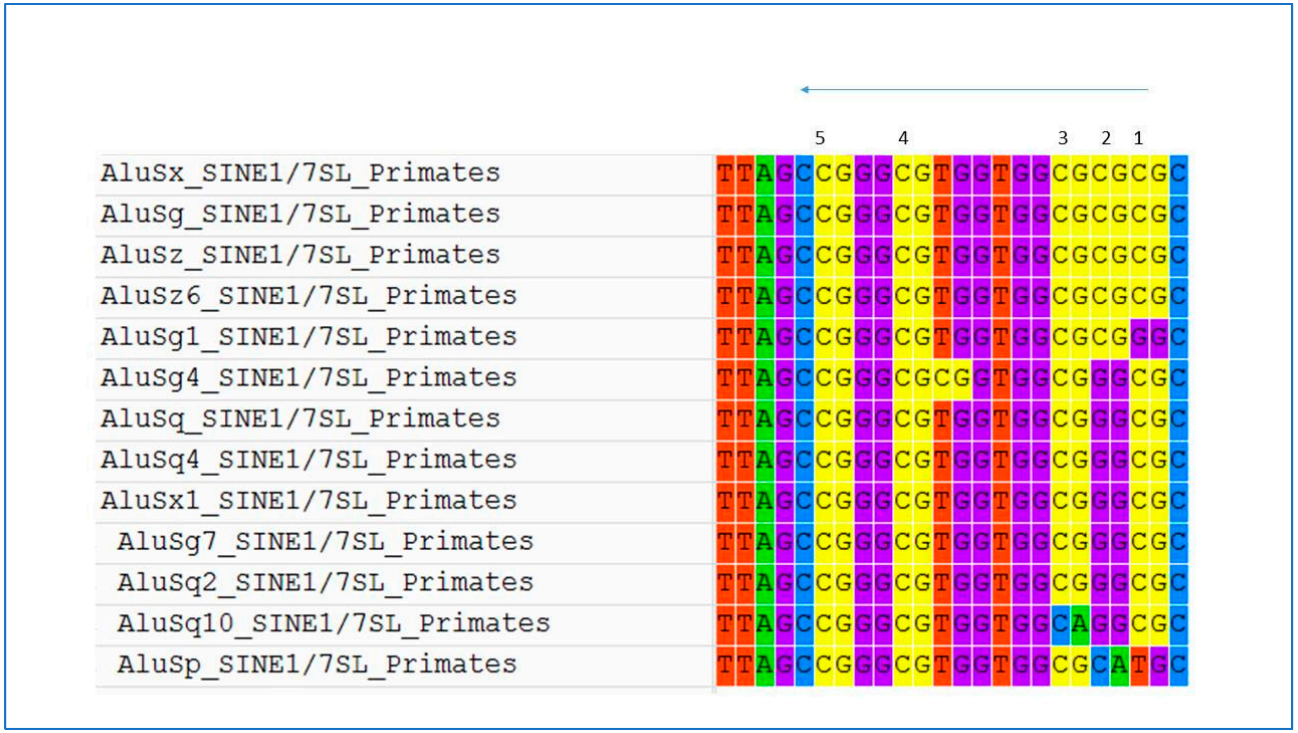

Figure 2S. Multiple sequence alignment of the consensus sequences of the target Alu subfamilies. The sequence portion here shown corresponds to positions 334-357 in the Alu Sx consensus, and contains the CpG sites (indicated by numbers from 1 to 5) analysed in the assay. The arrow represents the direction of the pyrosequencing. It can be noted that only part of the target subfamilies possesses all the 5 assayed CpGs.

Supplementary Table 2S. Alu bisulfite pyrosequencing: genomic distribution of target Alu subfamilies.

By using the CLC Biomedical Genomics Workbench software (Qiagen), the genomic positions of the Alu elements of the targeted subfamilies have been annotated with respect to known genes and regulatory regions (see table below). All annotations are referred to the human hg19 genome.

**Alu elements of target subfamilies [1]**			**Overlapping with genes[2]**	**Overlapping with exons[3]**	**Overlapping with regulatory regions[4]**
**Position with respect to genomic annotations**		Number (percentage)			
**Intragenic**	Intronic	329 955 (57.0%)	Yes	No	Not checked
	Exonic	12 418 (2.1%)	Yes	Yes	Not checked
**Intergenic**	Intergenic and NOT in regulatory regions	207 821 (35.9%)	No	No	No
	Intergenic and in regulatory regions	28 359 (4.9%)	No	No	Yes
**Total Alu elements in the targeted subfamilies**		578 553 (100%)			

Note [1] Elements of the Alu Sx, Sg, Sz, Sz6, Sg1, Sg4, Sq, Sq4, Sx1, Sg7, Sq2, Sq10 and Sp subfamilies, from Repeat Masker annotation track (based on RepBase definition of repeat elements subfamilies), on the human hg19 genome, downloaded from the UCSC Genome Browser Web site (http://genome.ucsc.edu); [2] "Homo Sapiens Ensemble v74 Genes" and [3]"Homo Sapiens Ensemble v74 mRNA" tracks downloaded from the Ensemble database (https://www.ensembl.org); [4] transcription factor binding sites track annotated in the ENCODE project ("wgEncodeRegTfbsClusteredV3"), downloaded from UCSC Genome Browser Web site (http://genome.ucsc.edu).

Supplementary Table 3S. Correlation Matrix from factor analysis of Alu CpGs

	ALU CPG 2	ALU CPG 3	ALU CPG 4	ALU CPG 5
**ALU CPG 1**	0.235*	-0.02	0.315*	0.246*
**ALU CPG 2**		0.778**	0.393**	0.558**
**ALU CPG 3**			0.436**	0.646**
**ALU CPG 4**				0.383**

* P < 0.05 ** p < 0.01

Supplementary Table 4S. Total variance explained from factor analysis of Alu CpGs

	Initial Eigenvalues			Extraction Sums of Squared Loadings			Rotation Sums of Squared Loadings		
Component	Total	% of Variance	Cumulative %	Total	% of Variance	Cumulative %	Total	% of Variance	Cumulative %
1	2.706	54.112	54.112	2.706	54.112	54.112	2.492	49.837	49.837
2	1.070	21.398	75.510	1.070	21.398	75.510	1.284	25.673	75.510
3	.626	12.511	88.021						
4	.453	9.063	97.084						
5	.146	2.916	100.000						

Extraction Method: Principal Component Analysis.

Supplementary Table 5S. Component Matrix[a] from factor analysis of Alu CpGs

	COMPONENT	
	1	2
**ALU CPG 1**	0.348	0.882
**ALU CPG 2**	0.86	-0.149
**ALU CPG 3**	0.865	-0.404
**ALU CPG 4**	0.665	0.323
**ALU CPG 5**	0.809	-0.054

[a] 2 components extracted. Extraction method: principal component analysis.

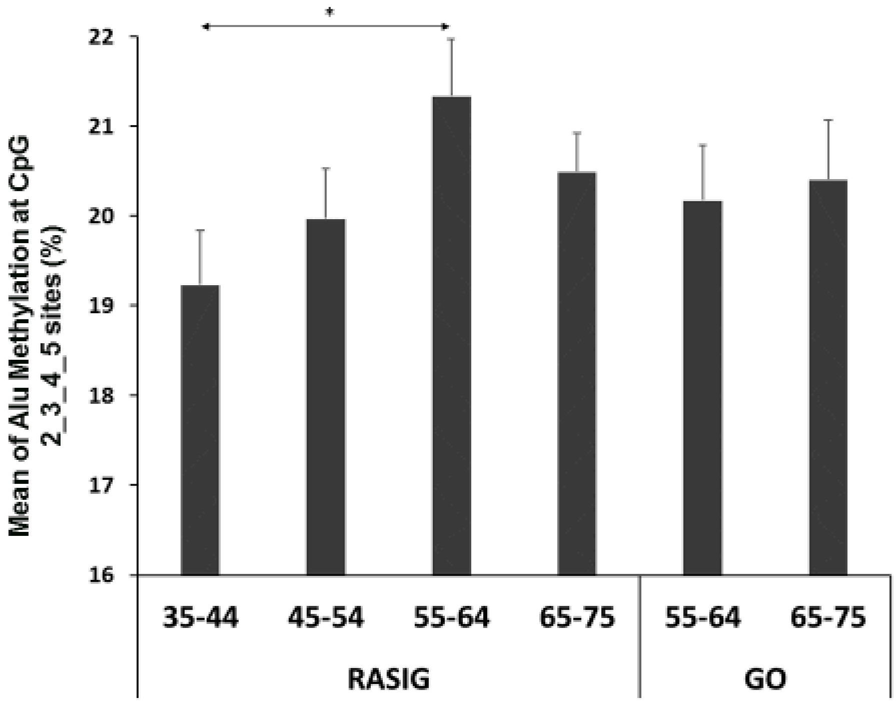

Figure 5S. Mean levels of Alu methylation at CpG2, CpG3, CpG4, CpG5, in DNA extracted from whole blood of RASIG (n. 60) and GO donors (n.32) recruited in Italy.

*p<0.05

Supplementary Table 6S. Automatic linear regression analysis for variables independently associated with mean levels of Alu methylation at CpG2, CpG3, CpG4 and CpG5 in RASIG and GO donors

Predictors	Coefficient	Std. Error	Importance	Sig
Meat consumption[a]	-2.189	0.682	0.361	0.002
Age	0.048	0.021	0.174	0.029
Brown bread consumption	2.599	1.210	0.161	0.035

[a]For meat consumption: =1 serv/day was compared to consumption of meat 2-7 serv/week (used as reference).

[b]For brown bread consumption: = 1-6 serv/week was automatically combined with ≥ 1 serv/day, used as reference and compared to < 1 serv/day.

References cited in this supplemental document.

Cui F, Sirotin MV, Zhurkin VB. Impact of Alu repeats on the evolution of human p53 binding sites. Biol Direct. 2011 Jan 6;6:2. doi: 10.1186/1745-6150-6-2.

Luo Y, Lu X, Xie H. Dynamic Alu methylation during normal development, aging, and tumorigenesis. Biomed Res Int. 2014;2014:784706. doi: 10.1155/2014/784706.

Jurka J, Kapitonov VV, Pavlicek A, Klonowski P, Kohany O, Walichiewicz J: Repbase Update, a database of eukaryotic repetitive elements. Cytogenet Genome Res. 2005, 110 (1-4): 462-467. 10.1159/000084979
